# Assessment of intracranial pressure with ultrasonographic retrobulbar optic nerve sheath diameter measurement

**DOI:** 10.1186/s12883-017-0964-5

**Published:** 2017-09-29

**Authors:** Dachuan Liu, Zhen Li, Xuxiang Zhang, Liping Zhao, Jianping Jia, Fei Sun, Yaxing Wang, Daqing Ma, Wenbin Wei

**Affiliations:** 1 Beijing Tongren Eye Center, Beijing Tongren Hospital, Capital Medical University; Beijing key Laboratory of Intraocular Tumor Diagnosis and Treatment; Beijing Ophthalmology and Visual Sciences Key Lab, Beijing, 100730 China; 2 Department of Ophthalmology,Xuanwu Hospital, Capital Medical University, Beijing, China; 3 Department of Neurology,Xuanwu Hospital, Capital Medical University, Beijing, China; 4 Beijing Geriatric Healthcare Center, Beijing, China; 5 Beijing Institute of Ophthalmology, Beijing, China; 6 Anaesthetics, Pain Medicine and Intensive Care, Department of Surgery and Cancer, Faculty of Medicine, Imperial College London, and Chelsea and Westminster Hospital, London, UK

**Keywords:** B-scan ultrasonography, Optic nerve sheath diameter, Intracranial pressure

## Abstract

**Background:**

Ultrasonograpic retrobulbar optic nerve sheath diameter (ONSD) measurement is considered to be an alternative noninvasive method to estimate intracranial pressure,but the further validation is urgently needed. The aim of the current study was to investigate the association of the ultrasonographic ONSD and intracranial pressure (ICP) in patients.

**Methods:**

One hundred and ten patients whose intracranial pressure measured via lumbar puncture were enrolled in the study. Their retrobulbar ONSD with B-scan ultrasound was determined just before lumber puncture. The correlation between the ICP and the body mass index (BMI), ONSD or age was established respectively with the Pearson correlation coefficient analysis. The discriminant analysis was used to obtain a discriminant formula for predicting ICP with the ONSD、BMI、gender and age. Another 20 patients were recruited for further validation the efficiency of this discriminant equation.

**Results:**

The mean ICP was 215.3 ± 81.2 mmH_2_O. ONSD was 5.70 ± 0.80 mm in the right eye and 5.80 ± 0.77 mm in the left eye. A significant correlation was found between ICP and BMI (*r* = 0.554, *p* < 0.001), the mean ONSD (*r* = 0.61, *P* < 0.001), but not with age (*r* = −0.131, *p* = 0.174) and gender (*r* = 0.03, *p* = 0.753). Using receiver operating characteristic (ROC) curve analysis, the critical value for the risk mean-ONSD was 5.6 mm from the ROC curve, with the sensitivity of 86.2% and specificity of 73.1%. With 200 mmH_2_O as the cutoff point for a high or low ICP, stepwise discriminant was applied, the sensitivity and specificity of ONSD predicting ICP was 84.5%-85.7% and 86.5%-92.3%.

**Conclusions:**

Ophthalmic ultrasound measurement of ONSD may be a good surrogate of invasive ICP measurement. This non-invasive method may be an alternative approach to predict the ICP value of patients whose ICP measurement via lumbar puncture are in high risk. The discriminant formula, which incorporated the factor of BMI, had similar sensitivity and higher specificity than the ROC curve.

## Background

Intracranial hypertension is a critical life-threatening condition caused by a variety of neurological and non-neurological diseases. It is also a sign of poor prognosis including risk of death from brainstem herniation [1]. Accordingly, it requires a rapid recognition to allow for the timely effective treatment [2]. Currently, a direct and invasive measurement via lumbar puncture is commonly used for ICP measurement clinically [3, 4]. However,potential risks such as hemorrhage, infection, and brainstem herniation with this invasive measurement of ICP are greatly concerned [5]. Furthermore, it is also not feasible to conduct on the patients who are too young or with contraindication such as coagulopathy, puncture area skin infection and thrombocythemia [6].

Recently, non-invasive techniques, such as transcranial Doppler sonography (TCD), tympanic membrane displacement (TMD), magnetic resonance imaging (MRI), cranial computed tomography (CT) or ultrasound have been utilized to assess the ICP [7–12]. In general, the optic nerve sheath diameter (ONSD) measurement is one of the parameters used for indirect prediction of ICP in these techniques. The optic nerve sheath (ONS) is continuous with meninges, and subarachnoid space. It has been shown that the pressure within the ONS is increased linearly with ICP increased [13].The ONSD was also found to be enlarged with the increased ICP [14]. This enlarged ONSD can be measured by non-invasive methods described above and the measurements have been found to correlate with direct ICP measurements with different techniques. [15–19] Indeed, ONSD measured with B-scan ultrasound exceeding a certain value can indicate high ICP that was well documented previously [11, 12, 20, 21]. Comparing with the CT and MRI, the ultrasound is much more convenient in particular coma patients and in emergency conditions, and also cheaper in costs. Caution to be taken is that previous studies including our own studies also indicated huge variations of the ONSD even in healthy adults [18, 22]. Therefore, we set to further re-evaluate the association of the ultrasonographic ONSD with ICP and to provide further evidence whether ONSD can be used for the indirect measurement of ICP clinically.

## Methods

**Fig. 1:**
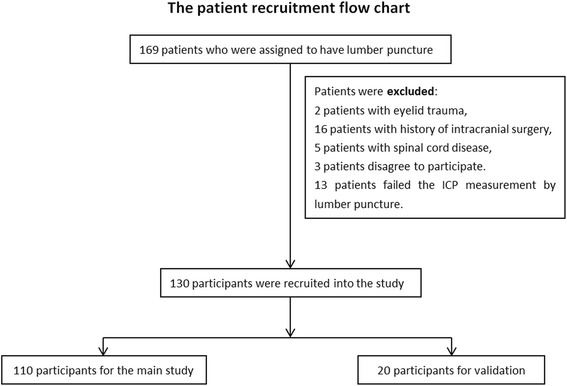
Patient recruitment flow chart

This is a hospital-based prospective observational study conducted in the Department of Ophthalmology and the Department of Neurology in Beijing Xuanwu Hospital from January 2011 to May 2012. After obtained Ethics Committee approval from Xuanwu Hospital and written informed consent, patients who underwent lumber puncture due to various neurological symptoms or diagnosis were enrolled into the study. Participants who had trauma and skin disease conditions in the eyelids that may influence B-scan ultrasound measurement were excluded. The exclusion criteria also were those who had a history of intracranial surgery or spinal cord disease, or who failed the ICP measurement by lumber puncture for any reasons. One hundred and thirty patients with informed consent participated this study (Fig. 1). In that, the first 110 participants were recruited for the main study and the next 20 patients were recruited for further study validation.

The ONSD at 3 mm behind the eyeball was measured with B-scan ultrasound. The ONSDs in both eyes were measured 3 times by an experienced operator who was blinded to the research protocol. The mean value of three measurements was recorded as an individual ONSD of the patient.

After the ultrasound examination, the ICP was measured by lumbar puncture within 10 min. Lumbar puncture was conducted under local anesthesia in a standardized manner in a lateral decubitus position, with the patient’s neck bent in full flexion and the knees bent in full flexion up to the chest. A standard spinal needle (20-gauge needle, 90 mm length) was inserted between lumbar vertebrate L3 ⁄ L4 or L4 ⁄ L5. The stylet from the spinal needle was withdrawn, a manometer was connected to the needle once cerebrospinal fluid dropping out, and the opening pressure of the CSF was measured to be ICP. All lumbar puncture and ICP measurement were performed between 8 to 10 AM. Patient’s height, body weight and body mass index (BMI) was measured and recorded.

The data was presented as mean ± standard deviation (SD). The Pearson correlation coefficient was analyzed for assessing the correlation between ICP and BMI, ONSD or age respectively. The discriminant analysis was used to obtain a discriminant formula for predicting ICP with ONSD、BMI、gender and age. All statistical analyses were performed with SPSS software (SPSS for Windows, version 16.0, SPSS, Chicago, IL, USA). A *P*-value less than 0.05 was considered to be of statistical significance.

## Results

**Fig. 2:**
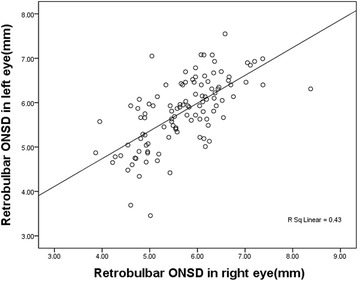
The correlation of retrobulbar optic nerve sheath diameter measured in the right eye and in the left eye. Scatter plot of ONSD shows good correlation between two eyes (*r* = 0.656, *P* < 0.001)

Of the 110 participants in test group, 58 (52.7%) were men and 52 (47.3%) were women, with the mean age of 38.3 ± 14.5 years (from 12 to 68 years). The mean BMI was 24.9 ± 4.2 kg/m^2^ and ICP measurement of 215.3 ± 81.2 mmH_2_O. The ONSD measurement by B-scan ultrasound was 5.70 ± 0.80 mm, 5.80 ± 0.77 mm, and 5.75 ± 0.71 mm, in the right side, left side, and the mean of both sides, respectively. All these data were summarized in the Table 1. The ONSD in the right side and in the left side was strongly correlated (*r* = 0.656, *p* < 0.001) (Fig. 2).

**Table 1:** Descriptive of participants’ information and main measurements

	minimum	maximum	Mean ± SD
Age (year)	12	68	38.3 ± 14.5
BMI (kg/m^2^)	17.7	35.5	24.9 ± 4.2
ICP (mmH_2_O)	50	450	215.3 ± 81.2
ONSD-R(mm)	3.87	8.38	5.70 ± 0.80
ONSD-L (mm)	3.46	7.55	5.80 ± 0.77
ONSD-mean (mm)	4.15	7.34	5.75 ± 0.71

ONSD-R: the optic nerve sheath diameter of the right eye measured with B-scan ultrasound;

ONSD-L: the optic nerve sheath diameter of the left eye measured with B-scan ultrasound;

ONSD-mean: the mean optic nerve sheath diameter of both eyes

**Fig. 3:**
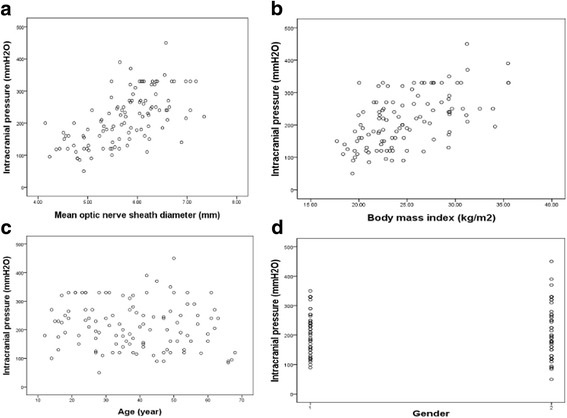
The scatter plot of intracranial pressure (ICP) and the mean optic nerve sheath diameter (ONSD-mean) of both eyes and intracranial pressure and the body mass index. ICP was significantly associated with the mean ONSD (*r* = 0.61, P < 0.001) (**a**) and BMI (*r* = 0.55, P < 0.001) (**b**), not associated with age (*r* = −0.131, *P* = 0.174) (**c**) or gender (*r* = 0.03, *P* = 0.753) (**d**). In Fig. D,1 is male, 2 is female

Using univariates analysis, ICP was significantly associated with the mean ONSD (*r* = 0.61, *P* < 0.001) (Fig. 3a) or BMI (*r* = 0.55, p < 0.001) (Fig. 3b), but not associated with age (*r* = −0.131, *P* = 0.174) (Fig. 3c) or gender (*r* = 0.03, *P* = 0.753) (Fig. 3d).

**Fig. 4:**
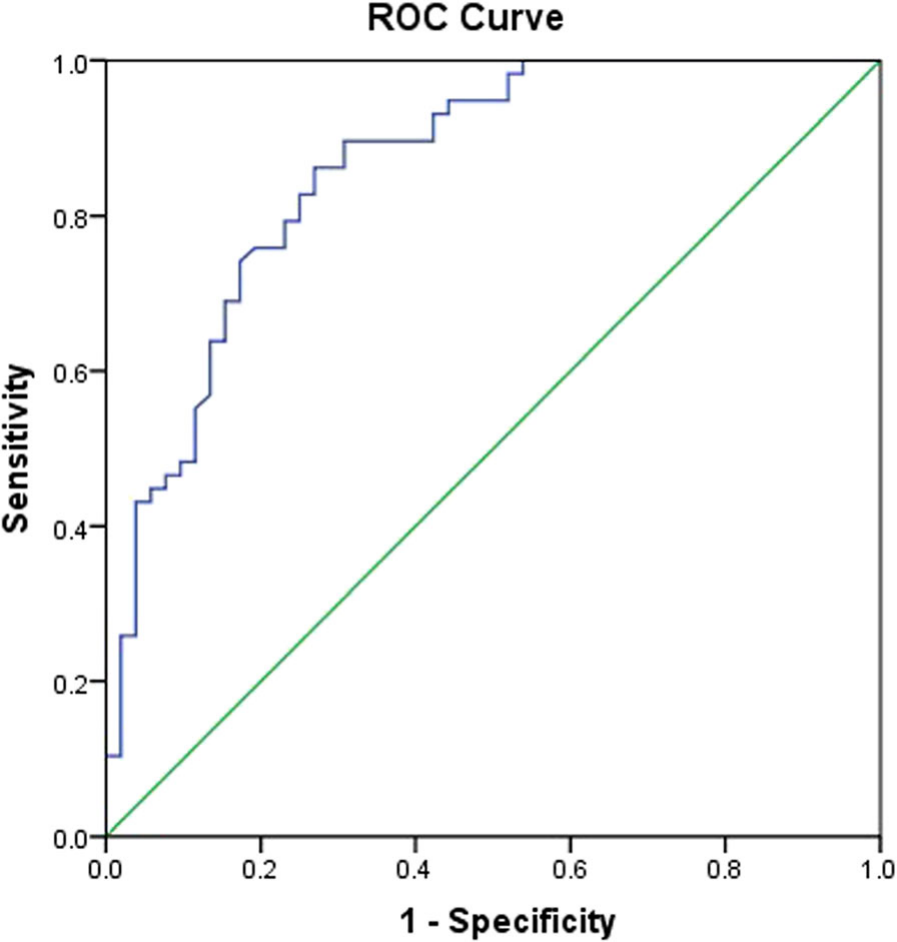
Receiver operating characteristic (ROC) curve for the mean-ONSD. The area under the curve (AUC) of 0.861 means that 86.1% of the time, a randomly selected individual who with intracranial hypertension has a wider mean-ONSD than that of patients who with normal intracranial pressure

The ROC for the mean ONSD had a high ability to discriminate between normal and high intracranial pressure, where the AUC value was 0.861 (Fig. 4); it was calculated that the critical value for the risk mean-ONSD was 5.6 mm from the ROC curve. If it is the case, then the sensitivity was 86.2% and specificity was 73.1%.

When a high ICP was defined as it is more than 200 mmH_2_O, there were 52 and 58 patients who had normal or high ICP respectively. Using discriminant analysis in a stepwise manner, the variables of age, gender, BMI, mean ONSD were taken as independent factors, a discriminant equation was obtained to be as: D = 0.169 × BMI + 1.484× mean ONSD-12.74. If the function value greater than 0, the patient was speculated to have high ICP. The Wilk’s lambda value of this function was 0.496 (Χ [2] =75.11, *p* < 0.001) with 85.5% of original grouped cases that has been correctly classified (Table 2). Based on the discriminant formula obtained above, 86.5% of cases with normal ICP and 84.5% of cases with high ICP were correctly classified (a total correction rate was 85.5%). The sensitivity is 84.5%, and the specificity is 86.5%.

**Table 2:** Classification results in the study group

	Group	Predicted Group Membership		Total
		Normal ICP group	High ICP group	
Original group	Normal ICP group	45 (86.5%)	7 (13.5%)	52
	High ICP group	9 (15.5%)	49 (84.5%)	58
Total		54	56	110

In order to test the efficiency of this discriminant equation, another 20 participants was collected as the validation group. Using the same formula obtained above, 92.3% of cases with normal ICP and 85.7% of cases with high ICP were correctly classified (a total correction rate 90.0%). The sensitivity is 85.7%, and the specificity is 92.3%. (Table 3).

**Table 3:** Classification results in validation group

	Group	Predicted Group		Total
		Low ICP group	High ICP group	
Original group	Normal ICP group	12 (92.3%)	1 (7.7%)	13
	High ICP group	1 (14.3%)	6 (85.7%)	7
Total		13	7	20

**Fig. 5:**
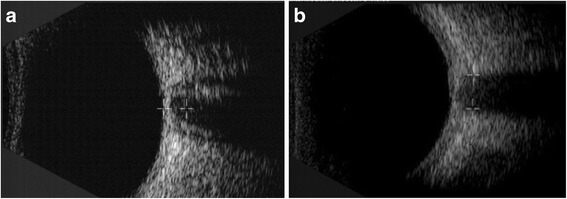
The scan images of ONSD measurement with B-scan ultrasound. **a** is the picture of ultrasonic measurement of ONSD in a high intracranial pressure patient. In this picture, there is a more significant subarachnoid space around optic nerve. **b** is the picture of ultrasonic measurement of ONSD in a normal intracranial pressure patient

Of note, it was found that in addition to having a wider ONSD, there is a significant increase of subarachnoid space around optic nerve in the ultrasonic scan in a high intracranial pressure patient (Fig. 5a) than in the normal intracranial pressure patient (Fig. 5b).

## Discussion

In our study, we found that ICP was significantly associated with the mean ONSD (*r* = 0.61, *P* < 0.001) and BMI (*r* = 0.55, *p* < 0.001). Using discriminant analysis, a discriminant equation was obtained as: D = 0.169 × BMI + 1.484 × mean ONSD-12.74. (Χ [2] =75.11, p < 0.001). The correction rate of this equation was 85.5%–90%. The method was with sensitivity of about 85%, and the specificity of 86.5%–92.3%.

Ultrasonography for measuring ONSD has been developed and its measurement has been suggested to be a possible indicator of intracranial hypertension. Hansen and Helmke used ultrasound in a cadaver study demonstrating that in the area just behind the eyeball, elevated pressure can increase the sheath diameter by more than 50% [23]. In an another study, the same group used intrathecal infusion test to prove that the human ONS has sufficient elasticity to allow a detectable dilation in response to intracranial hypertension and a liner relationship between ONSD and the cerebrospinal fluid (CSF) pressure was present within a certain range of CSF pressure [24]. Further, Tamburrelli and colleagues found that the ONS began to expand when the diastolic ICP was increased to be greater than 13–14 mmHg [25]. Beyond that point, a linear correlation was found between the enlargement of the ONSD and ICP. In clinical practice, ICP more than 15 mmHg (200 mmH_2_O) is considered to be intracranial hypertension. All these suggested that ophthalmic B-scan ultrasound can determine this enlarged ONSD and may indicate an increased ICP.

Additionally, several clinical reports showed that the ONSD enlargement was a sign of high ICP. For example, Rajajee V and colleagues conducted a prospective observational study in which patients were enrolled with either external ventricular drains or intraparenchymal ICP monitors at risk for intracranial hypertension in the intensive care unit [26]. A total of 536 ONSD measurements were performed on 65 patients. The results showed that optimal ONSD as no less than 0.48 cm for detection of high ICP (>20 mmHg) had a sensitivity of 96% (95% CI 91–99%) and a specificity of 94% (92–96%). Amini and colleagues also used the sonographic measurement of the ONSD and found that the ONSD of greater than 5.5 mm was a good indicator of high ICP (>20 cm H_2_O) with sensitivity and specificity of 100% (95% CI, 100–100) (*P* < 0.001) [20]. Maude and colleagues found that ONSD more than 4.75 mm should be considered to have a high ICP, according to the findings in 136 healthy Bangladeshi adults and children [27]. Dubourg and colleagues did a systematic review and meta-analysis composed of 231 patients from 6 studies and found the pooled sensitivity of 0.90, the specificity of 0.85, and the diagnostic odds ratio of 51, for the ONSD to detect raised ICP [12]. All these studies suggested that the ONSD measurement with ophthalmic ultrasound may be an effective method to assess ICP. However, we noticed that the cut-off value of ONSD varied from 4.75 mm to 5.9 mm. It is quite difficult to decide which value is more suitable, especially for Chinese population. In this group of patients, we found that the ONSD of greater than 5.6 mm was an indicator of high ICP (>200 mmH_2_O) with sensitivity of 86% and specificity of 73% which is in line with a previous study [20].

Previous studies showed that the ONSD measurement in the healthy population had a big range of variation. Bäuerle and colleagues measured the right ONSD of 15 healthy volunteers by both transbulbar sonography and 3 Tesla MRI. They found that the ONSD at 3 mm behind the eyeball was 5.43 ± 0.49 mm (4.6 ~ 6.4 mm), and 5.69 ± 0.77 mm (4.7 ~ 7.9 mm), by ultrasound and by MRI, respectively [18]. By comparing the ultrasound-derived and the MRI-derived ONSD values, it was found that the acceptable consistency between both methods was at a depth of 3 mm (*r* = 0.72, *P* = 0.002, mean difference < 5%). Our group also measured retrobulbar ONSD among a group of healthy Chinese adults with B-scan ultrasonography and the normal range of the ONSD at 3 mm behind eyeballs were 3.6 ~ 6.6 mm (mean ± SD 4.74 ± 0.62 mm) [22].However, the studies in those healthy volunteers were lack of the information about ICP. To clarify, we measured a group adults with normal ICP measurement [28]. In the current study, the ONSD at 3 mm behind the eyeball measured by B mode ultrasonography in adults with the normal ICP was 5.38 ± 0.67 mm (3.9 ~ 7.4 mm) in the right eyes, and 5.45 ± 0.73 mm(3.5 ~ 7.0 mm) in the left eyes. The ICP in this group of participants also had a variance from 5 to 15 mmHg, because there are many factors which affect ICP value including BMI value [28]. Indeed, Xie and colleagues found that the lumbar CSF pressure values were significantly associated with the BMI (*r* = 0.61; *P* < 0.0001) [29]. In this study, a significant positive correlation between ICP and BMI (*r* = 0.55, *P* < 0.001) was also found. Using stepwise method of discriminant analysis, the BMI was still an important factor that associated with ICP as reported. Taken together, further validation of ONSD and ICP either in healthy or neurological disease conditions is needed. In our study, the discriminant formula, which incorporated the factor of BMI, had similar sensitivity (84.5–85.7% and 86.2%) and higher specificity (86.5–92.3% and 73.1%) than the ROC curve.

Nevertheless, caution must be taken is that: first, ONSD measured with B ultrasound is an indirect method that can only predict a high or normal ICP. However, the exact value of ICP cannot be given. It can not replace the direct ICP measurement with the invasive method. In addition, there are about 5%–15% of the patients who were classified incorrectly. Second, even if the prediction of ICP with ONSD is accuracy, dynamic real time monitoring is not accessible.

## Conclusion

In summary, our data suggested that a significant correlation existed between ophthalmic ultrasound measurement of ONSD and ICP. By using the above discriminant equation, a good sensitivity and better specificity has been obtained in ICP assessment than using ROC curve. Although it cannot replace the invasive ICP measurement, this non-invasive method with ultrasound measurement of ONSD may be an alternative approach to predict the ICP value of patients whose ICP measurement via lumbar puncture are in high risk.

## Abbreviations

BMI: Body mass index
CT: Cranial computed tomography
ICP: Intracranial pressure
MRI: Magnetic resonance imaging
ONS: Optic nerve sheath
ONSD: Optic nerve sheath diameter
ROC curve: Receiver operating characteristic curve
TCD: Transcranial Doppler sonography
TMD: Tympanic membrane displacement
